# Supplementary material for: The interactome of intact mitochondria by cross-linking mass spectrometry provides evidence for coexisting respiratory supercomplexes
Source: Mol Cell Proteomics. 2017 Dec 8;17(2):216–32. doi: 10.1074/mcp.RA117.000470 (PMC5795388; doi:10.1074/mcp.RA117.000470)
Supplement: Supplemental Data [file supp_RA117.000470_133922_0_supp_23978_fzffwf.zip › spectra_annotation/mito_spectra_annotation/305-1-7-1-42-1.pdf]

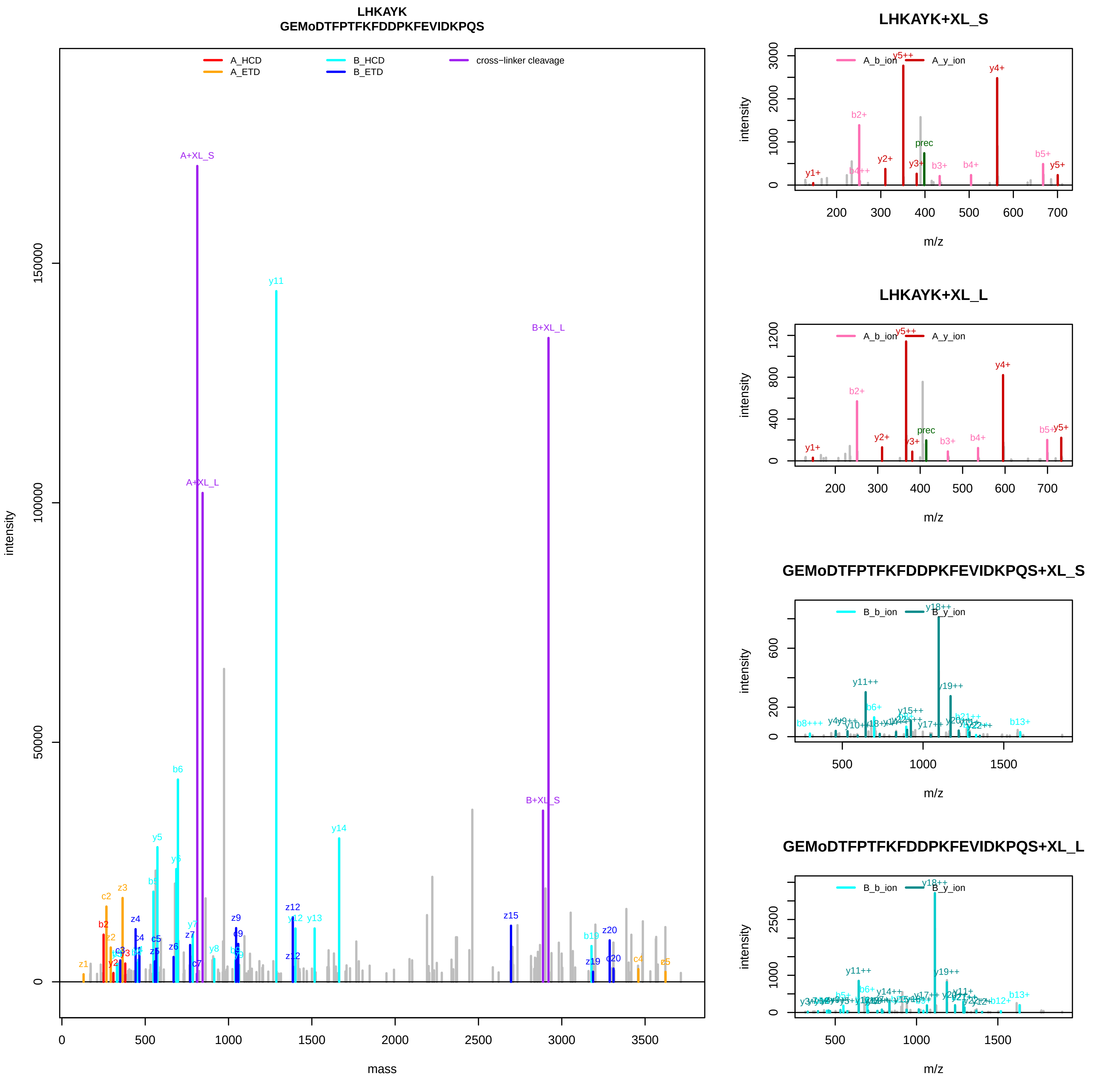

LHKAYK
GEMoDTFPTFKFDDPKFEVIDKPQS
A_HCD
A_ETD
B_HCD
B_ETD
cross−linker cleavage
A+XL_S
A+XL_L
B+XL_L
B+XL_S
y11
b6
y5
y14
z12
y13
z9
z15
z20
z19
c2
z3
b2
intensity
mass
LHKAYK+XL_S
LHKAYK+XL_L
GEMoDTFPTFKFDDPKFEVIDKPQS+XL_S
GEMoDTFPTFKFDDPKFEVIDKPQS+XL_L
A_b_ion
A_y_ion
B_b_ion
B_y_ion
prec
y5++
y4+
b2+
y2+
y3+
b3+
b4+
b5+
y5+
y1+
y18++
y11++
y19++
b13+
m/z
